# Tight Chk1 Levels Control Replication Cluster Activation in *Xenopus*

**DOI:** 10.1371/journal.pone.0129090

**Published:** 2015-06-05

**Authors:** Marie Platel, Arach Goldar, Jennifer M. Wiggins, Pedro Barbosa, Pierre Libeau, Pierre Priam, Hemalatha Narassimprakash, Xenia Grodzenski, Kathrin Marheineke

**Affiliations:** Department of Genome Biology, Institute for Integrative Biology of the Cell (I2BC), CNRS, CEA, Paris South University, Gif sur Yvette, France; University of Oxford, UNITED KINGDOM

## Abstract

DNA replication in higher eukaryotes initiates at thousands of origins according to a spatio-temporal program. The ATR/Chk1 dependent replication checkpoint inhibits the activation of later firing origins. In the *Xenopus in vitro* system initiations are not sequence dependent and 2-5 origins are grouped in clusters that fire at different times despite a very short S phase. We have shown that the temporal program is stochastic at the level of single origins and replication clusters. It is unclear how the replication checkpoint inhibits late origins but permits origin activation in early clusters. Here, we analyze the role of Chk1 in the replication program in sperm nuclei replicating in *Xenopus* egg extracts by a combination of experimental and modelling approaches. After Chk1 inhibition or immunodepletion, we observed an increase of the replication extent and fork density in the presence or absence of external stress. However, overexpression of Chk1 in the absence of external replication stress inhibited DNA replication by decreasing fork densities due to lower Cdk2 kinase activity. Thus, Chk1 levels need to be tightly controlled in order to properly regulate the replication program even during normal S phase. DNA combing experiments showed that Chk1 inhibits origins outside, but not inside, already active clusters. Numerical simulations of initiation frequencies in the absence and presence of Chk1 activity are consistent with a global inhibition of origins by Chk1 at the level of clusters but need to be combined with a local repression of Chk1 action close to activated origins to fit our data.

## Introduction

To maintain genome stability, eukaryotic DNA replication must be strictly controlled in space and time during S phase [1]. In higher eukaryotes, DNA replication starts from several thousand replication origins, each activated at different times during S phase. It also involves the coordinated activation of several replicons, or replicon clusters [2,3]. Recent genome-wide studies have shown that large segments of the genome—called replication domains—replicate together [4]. It is not clear how ordered origin activation at these different levels of chromosome organization is controlled. Assembly of the pre-replicative complex (pre-RC) during G1 phase at origins is initiated by binding of the origin recognition complex (ORC) to DNA sequences—this, in turn, recruits Cdc6, Cdt1 and the MCM 2–7 complex. The pre-RCs are subsequently activated at the G1/ S phase transition by Cyclin- and Dbf4-dependent kinases (CDKs and DDKs). CDKs and DDKs function to recruit additional factors that unwind DNA and start DNA synthesis at the origins. In higher eukaryotes, replication timing is controlled by Cyclin E/Cdk2 in the *Xenopus in vitro* system [5] and by Cyclin A/Cdk1 in human cells [6].

The spatio-temporal replication program is also controlled by the replication checkpoint that is activated in response to a threshold level of stalled replication forks or damaged DNA [7,8]. In the yeast *Saccharomyces cerevisiae*, this checkpoint depends on Mec1 and Rad53. It stabilizes stalled replication forks [9,10] and prevents or delays firing of late origins in the presence of stalled forks or DNA damage [11,12]. In sperm nuclei replicating in *Xenopus* egg extracts, forks stalled by the DNA polymerase inhibitor aphidicolin cause helicase to uncouple from polymerase activities. This generates large amounts of single-stranded DNA to which RPA and polα bind [13] which, together with primed DNA, generates the signal to activate ATR [14,15]. ATR phosphorylates and activates Chk1 [16,17], which in turn phosphorylates the phosphatase Cdc25A, leading to its degradation [18]. Cdc25A is required for Cyclin-Cdk2 activation [19]. Recent studies underlined the role of these checkpoint proteins during normal S phase for preventing replication stress in the absence of induced fork stalling and DNA damage. We and others have shown that ATR regulates origin firing during unchallenged S phase progression [20,21] but the role of Chk1 is unclear in early *Xenopus* embryos. Down regulation of XChk1 in early *Xenopus* embryos indicates that XChk1 is not vital during the first twelve cell divisions [22] and no effect of Chk1 depletion was detected on DNA replication in the *Xenopus in vitro* system in the presence of aphidicolin [23]. But Chk1 depletion accelerates mitosis entry in the ATR dependent S/M checkpoint [24]. In asynchronous mammalian cells, Chk1 inhibition by UCN-01 and Chk1 depletion led to increased origin density [25], reduced fork speed [26] and induced double strand breaks and DNA damage response [27]. Chk1 is a haplo-insufficient tumor suppressor [28] and is frequently overexpressed in lymphoma and breast carcinomas [29,30]. owever, it is not known whether Chk1 overexpression can affect replication origin activation in higher eukaryotes.

In early *Xenopus* embryos, S phase is brief and replication initiates without any sequence specificity [31]. Completely random distribution of origins would generate some unacceptable large inter origin distances to complete S phase in time. We and others have shown that replication origins are spaced 5 to 15 kb apart in the *Xenopus in vitro* system, and are clustered in early- and late-firing groups of origins (clusters) [20,32,33]. Replication timing is stochastic at the level of origins and clusters, but deterministic at the level of replication foci [34]. To understand the mechanisms that ensure complete DNA replication we proposed a numerical model for the control of DNA replication in *Xenopus* [35]. This model combines time-dependent changes in the availability of a replication factor and a fork-density dependent affinity of this factor for potential origins which explained best the observed increase in the initiation rate and fork density in our system. This model also fits with a very similar increase of replication frequency in yeast and humans [36], illustrating the universal character of our model. One open question is how the replication checkpoint inhibits origin firing in late clusters whereas origin activation in early clusters is still permitted. In this study we address this question by combining new DNA combing data of origin activation after modulating Chk1 levels and numerical simulations in the presence and absence of Chk1 kinase activity in the synchronous *Xenopus in vitro* system.

By specific inhibition using UCN-01 and AZD-7762 or immunodepletion of Chk1 we show that Chk1 regulates the spatio-temporal replication program at the level of replication clusters and not inside active clusters—both in the presence and absence of external replication stress. We show that Chk1 inhibition results in an increase in initiations in S phase in the absence and presence of aphidicolin, consistent with studies in mammalian cells. Surprisingly, modest Chk1 overexpression by adding recombinant Chk1 inhibits DNA replication by decreasing fork density and inhibiting cluster activation showing for the first time that Chk1 levels must be tightly controlled in our system to allow correct origin activation even in the absence of external stress. The numerical simulation of initiation frequencies in the presence and absence of checkpoint activity, and subsequent fitting to our experimental data, shows that Chk1 globally inhibits replication clusters whereas Chk1 is itself inhibited close to activated origins in active early clusters. Thus, we provide for the first time a numerical model for the spatio-temporal replication program including the replication checkpoint for higher eukaryotes.

## Materials and Methods

### Reagents and antibodies

Aphidicolin and UCN-01 were purchased from Sigma-Aldrich, AZD-7762 from Selleck Chemicals, aliquoted at -20°C and used only once, Human Anti-Phospho-Serine345-Chk1 (recognizes Phospho-Ser344-XChk1) was purchased from Cell Signaling Technology, anti-human Chk1 antibody from SantaCruzBiotech, anti-Phospho (Y15) cdk2 (ab76147) from Abcam, Anti-DNA antibody (Mab3032) from Merck-Millipore, Streptavidin and AlexaFluor antibodies from Invitrogen. XOrc2 antibody was a gift from R. A. Laskey.

### Production of antibody against XChk1 and recombinant XChk1

XChk1 cDNA (gift from B. Dunphy) was cloned into a pDEST vector (Invitrogen) including an N-terminal Histag. The protein was expressed in *E*.*coli* C41 (DE3) (gift of B. Miroux) and purified using Ni-Sepharose (GE Healthcare) according to the manufacturer. Two specific polyclonal antibodies against the full length recombinant protein were produced by P.A.R.I.S antibodies (Compiegne, France). These antibodies worked well in western blot analysis but did not work in immunodepletions experiments. For depletion and add back experiments recombinant and active XChk1 with a N-terminal His-tag was expressed in the baculovirus expression system (BD BaculoGold), purified using Nickel-Sepharose (Amersham Bioscience) beads as described by the supplier and dialyzed over night against 50 mM Hepes pH 7.8, 10% glycerol, 1mM DTT, 300mM KCl. Its kinase activity was tested using the Cdc25 peptide substrate CHKtide (Upstate) as indicated by the supplier.

### Replication of sperm nuclei in *Xenopus* egg extracts

Replication competent extracts from unfertilized *Xenopus* eggs were prepared as described [37] and used fresh unless stated otherwise. We routinely checked for Chk1 phosphorylation before nuclei addition in order to exclude low quality extracts. Sperm nuclei (100 or 2000 nuclei/μl) were incubated in extracts in the presence of cycloheximide (250 μg/ml), energy mix (7.5 mM creatine phosphate, 1 mM ATP, 0.1 mM EGTA, pH 7.7, 1 mM MgCl_2_) and 20μm biotin-dUTP (Roche Applied Science). Replication was allowed to continue for indicated time points. Aphidicolin was added at 7.5 μg/ml and replication continued for 90 to 120 min. UCN-01 (or solvent (DMSO) alone as control) was added at 1 μM. Caffeine (or buffer alone as control) was added where indicated, to a final concentration of 5 mM from a 100 mM solution, freshly dissolved in 10 mM Pipes-NaOH, pH 7.4. *In vitro* fertilization of *Xenopus* eggs with sperm was performed according to standard techniques [38], and developmental stages of embryos were determined according to Nieuwkoop and Faber (1994). Our institutional Animal Care and Use Committee (IACUC) namely Paris Center and South number 59 approved the study and the protocols herein (approvals number 2012–0062 and 2012–0063) following the French and the European laws on animal experimentation.

### Immunodepletions

Anti-XChk1 serum [24] or mock serum (rabbit IgG) was incubated 3h or overnight at 4° C with native protein A sepharose beads (GE Healthcare). Beads were washed with EB buffer without DTT buffer and briefly with a small volume of fresh extract to eliminate buffer and incubated twice 30 min at 4°C with egg extract (volume ratio 1:2) under agitation. Extracts were separated from beads by centrifugation for 2 min at 1000 g in compact reaction columns (USB) with cellulose filters and used for replication reactions.

### Molecular combing and detection by fluorescent antibodies

DNA was extracted and combed as described [39]. Biotin was detected with AlexaFluor594 conjugated streptavidin followed by anti-avidin biotinylated antibodies. This was repeated twice, then followed by anti-DNA antibody, AlexaFluor488 rabbit anti-mouse, and goat anti-rabbit antibodies for enhancement [40].

### Measurements and data analysis

Images of the combed DNA molecules were acquired and measured as described [39]. For each combing experiment a total of 6–12 Mb DNA was measured. The fields of view were chosen at random, unless mentioned otherwise. Measurements on each molecule were made using Image Gauge version 4.2 (Fujifilm) and compiled using macros in Microsoft Excel (2010). Replication eyes were defined as the incorporation tracks of biotin–dUTP. Replication eyes were considered to be the products of two replication forks, incorporation tracks at the extremities of DNA fibers were considered to be the products of one replication fork. Tracts of biotin-labeled DNA needed to be at least 1 kb to be considered significant and scored as eyes. When label was discontinuous, the tract of unlabeled DNA needed to be at least 1 kb to be considered a real gap. The replication extent was determined as the sum of eye lengths divided by the total DNA length. Fork density was calculated as the total DNA divided by the total number of forks. The midpoints of replication eyes were defined as the origins of replication. Eye-to-eye distances (ETED), also known as inter-origin distances, were measured between the midpoints of adjacent replication eyes. The means of fiber lengths were comparable inside each individual experiment in order to avoid biases in eye to eye distances. Incorporation tracks at the extremities of DNA fibers were not regarded as replication eyes, but were included in the determination of the replication extent, calculated as the sum of all eye lengths (EL) divided by total DNA. Box plots of ETED (with n ranging from 80–400) were made using GraphPad version 6.0 (La Jolla, CA, USA). Statistical analysis of repeated experiments have been included as means including standard error of the mean (SEM). Non parametric unpaired tests (Mann-Whitney Test) and unpaired Student’s t-tests were used to determine statistical significance. A P-value less than 0.05 was considered statistically significant. When experiments were repeated with a different egg extract replication extent differs at identical time scales because different egg extracts replicate nuclei with different replication kinetics. It is therefore difficult to combine all of them and include statistics of independent kinetics experiments.

### Neutral and alkaline agarose gel electrophoresis

Sperm nuclei were incubated in fresh extracts complemented with indicated reagents and one-fiftieth volume of [α-^32^P]dATP (3000 Ci/mmol). DNA was purified, separated on 0.8% TBE- agarose or 1.1% alkaline agarose gels, and analyzed as described [33].

### Western blot analysis

For analysis of whole extract samples, replication reactions were stopped at indicated times by the addition of SDS sample buffer. For analysis of nuclei, reactions were diluted into a 20-fold volume of nuclear isolation buffer (NIBS) (50 mM Hepes, 150 mM NaCl, 2 mM MgCl_2_, protease inhibitors, phosphatase inhibitors, 10% sucrose) and nuclei were pelleted through a NIBS buffer with 20% sucrose at 4000 g, 5 min, 4°C. The purification was repeated, then the pellet was dissolved in SDS sample buffer. For analysis of chromatin-bound proteins, reactions were diluted into a 20-fold volume of nuclear isolation buffer (NIB) (50 mM Hepes pH 7.5, 100 mM NaCl, 2 mM MgCl_2_, 2 mM DTT, spermin 0.2 mM, spermidine 0.5 mM, protease inhibitors, phosphatase inhibitors, 0.1% TritonX100) and chromatin was recovered through a NIBS buffer, 0.1% TritonX100, 15% sucrose at 4000 g, 5 min, 4°C. Interphase was washed twice with 200 μl NIB+ TritonX-100. The pellet was centrifuged again at 10 000 g for 5 min, 4°C and was resuspended in SDS sample buffer. Proteins were subjected to SDS gel electrophoresis and transferred to PVDF membranes. Immunodetection was performed according to the manufacturer, and peroxidase activity was revealed using Super Signal West Pico or Femto Chemiluminescence Kit (Pierce).

### Cdk2 immunoprecipitation and kinase assays

Anti-Xenopus Cdk2 antibody or mock rabbit IgG were coupled to Protein A Sepharose as described above and washed in dilution buffer (50mM Hepes/KOH pH8.0, 50mM KCl, 20 mM K_2_HPO_4_/KH_2_PO_4_ pH8). Replication reactions (50μl) supplemented with 2000 nuclei/μl were stopped after 45 min with 5 fold dilution buffer, proteinase and phosphatase inhibitors, overlayed on 150μl dilution buffer and 30% Sucrose and centrifuged 5000 g for 5 min. The pellet was resuspended in 200μl dilution buffer supplemented with 0.2% Triton X100 to extract nuclear proteins, incubated 10 min on ice and centrifuged 14 000 rpm for 5 min. The supernatant was incubated with Cdk2 or mock coupled beads at 4°C for 2 h. Beads were washed three times in dilution buffer with Triton, once in dilution buffer without Triton and finally in EB buffer. H1 histone kinase assays in duplicates were performed with 10 μl beads, 0.1 μCi γ^32^P-ATP, 50μM ATP and 0.5 μg H1 histone for 30 min at 30°C. Reactions were stopped with 2x Laemmli buffer, proteins were separated by SDS gel electrophoresis, gels were dried and bands were quantified on a phosphoimager Typhoon Trio (GE Healthcare).

### Numerical simulation of initiation frequency I(f)

We used a dynamic Monte Carlo method to simulate DNA replication as a one-dimensional nucleation and growth process [35,41]. The replicating genome is schematised as a one-dimensional array of *L* elements (L = 1000000 here). Each element corresponds to 1 kb. We made the following assumptions: 1. The initiation process is governed by the stochastic encounter of a limiting factor (*N*) and a potential replication origin; 2. The number *N* of limiting factors increases with a rate *J* as replication progresses (*N = N*_*0*_ *+Jt*, where *N*_*0*_ is the initial number of limiting factors); 3. Replication origins are uniformly distributed along the genome and can only fire once during the simulation. Once an initiation has occurred, the limiting factor is sequestered by the two diverging replication forks; replication forks will progress with a speed v = 1 element per round of calculation. Each round of calculation corresponds to 2 min, so the measured speed v of replication forks is 0.5 kb min^-1^. The encounter between a limiting factor and a replication origin will trigger firing with a probability *P(x*,*t)*, where *x* is the location of replication origin and *t* is the elapsed time from the beginning of S phase. *P(x*,*t)* represents the probability of replication origins firing, which is conditional on the inhibitory action of Chk1. As helicases are integrated parts of replication forks, in our numerical simulations we assumed that the number of activated Chk1 (*N*_*Chk1*_) is equal to the number of active replication forks. We checked by quantitative western blotting that the amount of chromatin bound Chk1 is roughly is sufficient for one Chk1 molecule to bind to one replication fork (data not shown). Furthermore, as the inhibitory action of Chk1 is thought to be global over the genome, we assumed that during an unchallenged S phase, Chk1 inhibits unfired potential replication origins with a probability *k*_*Chk1*_. Also, we assumed that in the absence of Chk1, the encounter between a limiting factor and a replication origin will trigger firing with a constant probability *P*_*0*_ if the latter has not been inhibited for firing by Chk1. We introduced a third, local mechanism that removes the inhibitory action of Chk1 over the potential replication origins. Assuming that this mechanism is also active during an unchallenged S phase, we considered that if a Chk1-inhibited potential replication origin is at a distance *d* = 45 kb (cluster size) of a replication fork, it would have a probability *k*_*polo*_ of recovering its ability to fire. Combining our numerical simulation with a Simplex optimisation algorithm, we obtained the fit of *I(f)* with experimental data.

## Results

### Global origin inhibition after induced fork stalling is Chk1-dependent in *Xenopus* egg extracts

**Fig 1:**
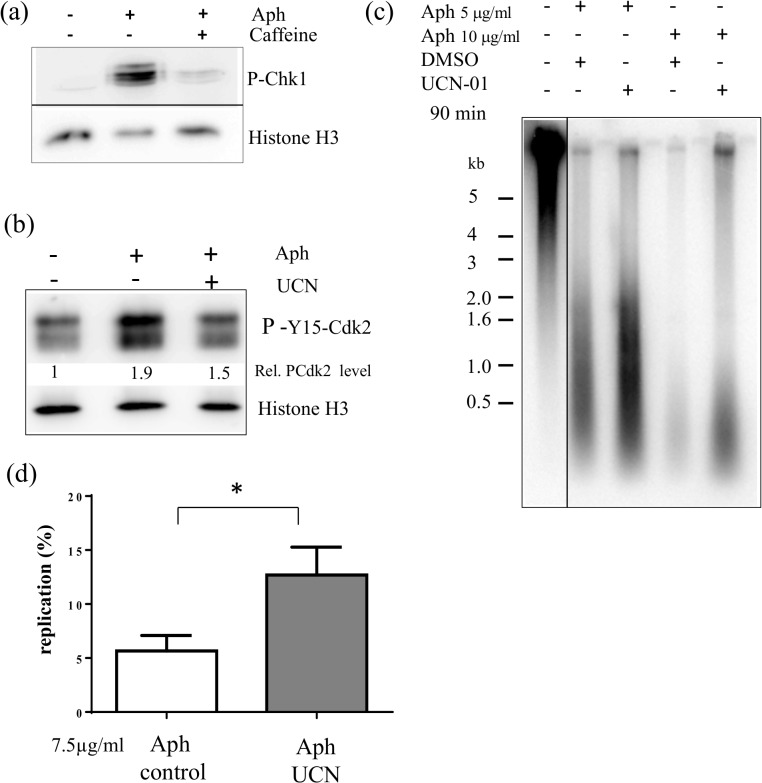
Inhibition of Chk1 activity by UCN-01 increases DNA synthesis in the presence of aphidicolin. (a) Sperm nuclei (1000n/μl) were added to egg extracts for 35 and 60 min in the presence or absence of aphidicolin and caffeine, and whole extracts were subjected to gel electrophoresis and western blot analysis using an anti-P-S345-Chk1 antibody, (b) Sperm nuclei were added to egg extracts for 90 min in the presence or absence of aphidicolin and 1μM UCN-01, western blot against whole extracts using antibody against phospho-Y15 Cdk2, loading control histone H3, (c) Sperm nuclei were added to egg extracts in the presence of [α-^32^P]-dATP with or without 1μM UCN and aphidicolin (5 or 10 μg/ml) and nascent DNA strands synthesized after 90 min were analyzed by alkaline DNA electrophoresis, (d) Quantification of six independent alkaline DNA electrophoresis experiments at 7.5 μg/ml aphidicolin, mean with SEM (t-test, P = 0.0387), 100% replication is defined as the signal in the–Aph-control after 90 min, * marks significant difference (P < 0.05).

We used nascent strand analysis and DNA combing to analyze origin activation of sperm nuclei replicating in the *Xenopus* egg extracts in the presence of aphidicolin and in the presence or absence of the specific Chk1 inhibitor UCN-01 as well as after Chk1 depletion. When sperm chromatin is incubated in *Xenopus* egg extract, it is assembled into normal interphase nuclei surrounded by a nuclear envelope and replicated semi-conservatively [42]. DNA replication starts synchronously after a typical 20 to 30 min lag phase, and is complete 40 to 60 min later, depending on the extract. Aphidicolin, an inhibitor of DNA polymerases α,ε and δ, inhibits elongation of DNA nascent strands, and thereby stalls replication forks, but does not prevent the initiation of DNA replication and the formation of short nascent strands. Upon addition of aphidicolin to the replication reaction, we observed a caffeine sensitive phosphorylation of Chk1 in western blot analysis from whole replication reaction samples using a specific phospho-Ser345 Chk1 antibody (Fig 1A), consistent with previous studies [21,23]. Next, we checked whether UCN-01, a specific inhibitor for Chk1 in mammalian cells [43,44], inhibits Chk1 in *Xenopus* egg extracts. Using western blot analysis with an antibody against the inhibitory Y15 phosphorylation site of Cdk2, we confirmed that the P-Cdk2 signal decreased in comparison to the aphidicolin control and that therefore the Chk1-Cdc25-Cdk2 axis was also abrogated by 1 μM UCN-01 in the *Xenopus in vitro* system in the presence of aphidicolin (Fig 1B). We analyzed the effect of UCN-01-mediated Chk1 inhibition on nascent strand synthesis by alkaline DNA electrophoresis. Sperm nuclei were incubated for 90 min in the presence of 5 or 10 μg/ml aphidicolin and [α-^32^P]-dATP with or without UCN-01. We expected that inhibition of Chk1 would increase nascent strand synthesis due to unscheduled origin firing if Chk1 inhibits origin activation in the replication checkpoint pathway. We observed that UCN-01 increased indeed the accumulation of nascent strand synthesis 2- and 5-fold at 5 and 10 μg/ml aphidicolin, respectively (Fig 1C). Thus, the inhibitory effect of Chk1 on replication increased with the dose of aphidicolin. Nascent strands were smaller with 10 μg/ml aphidicolin than with 5 μg/ml, owing to a stronger elongation inhibition, and were, in both cases, not affected by Chk1 inhibition. We repeated this experiment six times with very similar results but with an intermediate aphidicolin concentration (7.5 μg/ml) which allowed optimal DNA combing analysis (see below). We found a significant 2.2 fold mean increase upon UCN-01 addition (Fig 1D).

**Fig 2:**
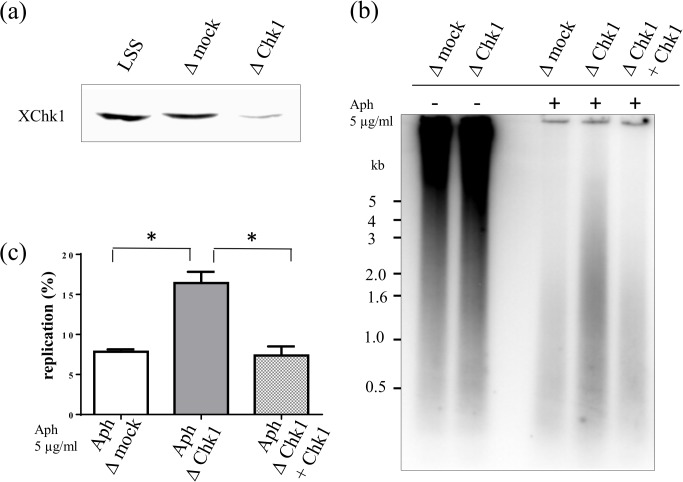
Chk1 depletion increases DNA synthesis in the presence of aphidicolin. (a) Egg extracts were mock- or Chk1-depleted and aliquots analyzed by western blotting using an anti-XChk1 antibody, (b) Sperm nuclei were incubated for 150 min in mock-depleted, Chk1-depleted egg extracts, Chk1-depleted egg extracts supplemented with recombinant 40 nM XChk1 in the presence of [α-^32^P]-dATP with or without aphidicolin (5 μg/ml). Nascent DNA strands synthesized after 150 min were analyzed by alkaline gel electrophoresis, (c) Mean replication in mock depleted, Chk1 depleted, Chk1 depleted + Chk1 add back extracts of two independent experiments with SEM (t-tests: mock versus Chk1 depletion: P = 0.026, Chk1 depletion versus add back: P = 0.037), * significantly different (P < 0.05).

In order to further confirm the role of Chk1 we performed immunodepletion experiments of Chk1. Using a specific *Xenopus* anti-Chk1 antibody [24] we depleted 85% of endogenous XChk1 in egg extracts (Fig 2A). Sperm nuclei were then incubated in the presence of 5 μg/ml aphidicolin and [α-^32^P]-dATP in mock or XChk1 depleted extracts. The experiment was repeated once. We found that nascent strand synthesis under denaturing conditions was twofold higher in XChk1-depleted extracts in comparison to mock depleted extracts (Fig 2B and 2C), consistent with our experiments with Chk1 inhibitors. Adding back recombinant active XChk1 (40nM, [24]) to XChk1-depleted extracts decreased DNA synthesis to control levels, which demonstrated the specificity of our immunodepletion. We conclude that Chk1 is activated and regulates origin firing upon fork stalling by aphidicolin in *Xenopus* egg extracts.

**Fig 3:**
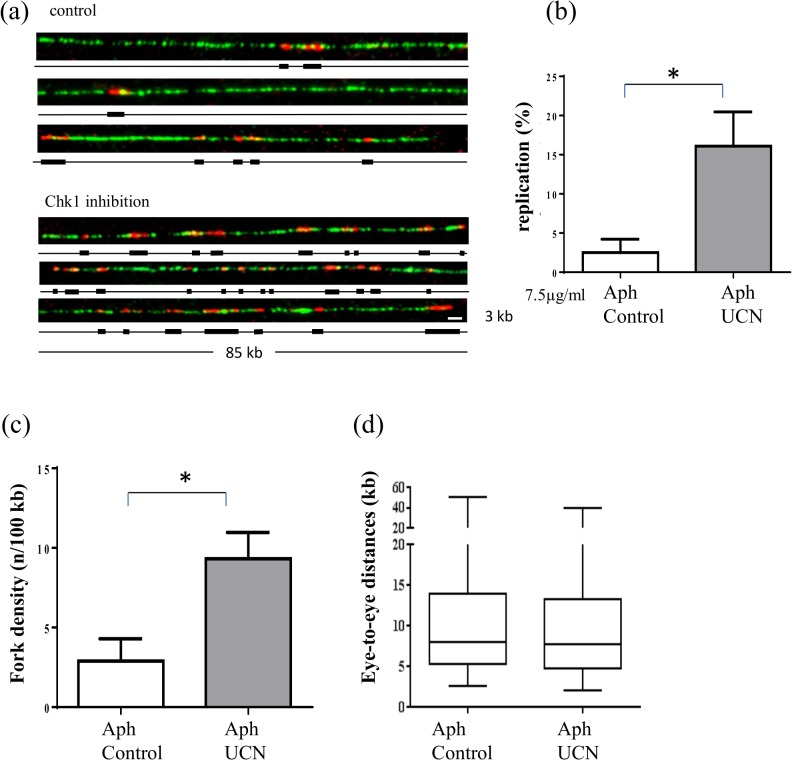
Fork density increases after Chk1 inhibition in the presence of aphidicolin induced stalled forks. Sperm nuclei (2000 nuclei/μl) were replicated in egg extracts in the presence of Biotin-dUTP, aphidicolin (7.5μg/ml) and in the presence (1μM) or absence of UCN-01 for 90 min. (a) Three representative combed DNA fibers replicated in the absence (above) or the presence of UCN-01 (below) (merge: green, whole DNA label; red, biotin labeled replication eyes), (b) Mean replication extent of four independent experiments with SEM (t-test, P = 0.027), (c) Mean fork density (number of forks/100kb) of four experiments with SEM (t-test, P = 0.018), (d) Box-plot of distances between replication eyes (kb) of representative experiment from (a), scale bar 3 kb,* significantly different (P < 0.05).

We performed DNA combing experiments to understand how Chk1 regulates origin firing in the presence of replication stress. Sperm nuclei were incubated in the presence of 7.5 μg/ml aphidicolin and DMSO or UCN-01 for 90 min in egg extracts. To label replication eyes, biotin-dUTP was added at the beginning of the reaction which was stopped after 90 min. DNA was purified, combed and labeled as described in the experimental procedures. DNA fibers were visualized using an anti-DNA antibody, replication eyes were visualized using fluorescent streptavidin conjugates (Fig 3A) and replication extent was determined. The mean replication extent of four independent experiments is shown in Fig 3B. We found that in the presence of aphidicolin the mean replication extent was around 6-fold higher in the presence of UCN compared to the control. In *Xenopus*, 2–5 origins are grouped in replication clusters (30–50 kb) that fire asynchronously throughout S phase. The increase of replication extent can result therefore from an increase in the number of origins activated either inside or outside already activated replication clusters, or both. To determine which origins are activated, we directly measured eye-to-eye distances on individual fibers. In addition, we calculated the overall fork density (number of forks/100 kb) by dividing the total DNA length by the total number of forks. Because DNA fibers analyzed by DNA combing are in general not longer than 80–100 kb due to DNA breaks a difference exists between fork density and eye-to-eye distances, especially in early S phase. Eye-to-eye distances can only be measured on fibers containing at least two origins, whereas the calculation of fork density also includes those fibers with only one origin, or no origins and therefore include all replication clusters which have not yet been activated. Therefore local eye-to-eye distances mainly reflect the origin distances from origins inside the same replication cluster, whereas fork density is representative of the amount of active origins in the whole population of DNA fibers. Thus, an increase of the fork density with no change in eye-to-eye distances would reflect an increase in replication cluster activation. We observed a mean 2.6-fold increase in fork density in aphidicolin-treated extracts when Chk1 was inhibited (Fig 3C). However, there was no significant decrease in eye-to-eye distances when Chk1 was inhibited (Fig 3D, median 8.1 kb control versus 7.8 kb plus UCN-01, Mann-Whitney, two-tailed test, P = 0.370), which would have been expected if additional origins fired inside active clusters. No significant difference was detected in eye-to-eye distributions in another independent experiment (S1 Fig). We conclude that when replication forks are stalled by aphidicolin, a Chk1 dependent replication checkpoint is activated in the *Xenopus in vitro* system, which inhibits origins outside, but not inside, activated clusters.

### Chk1 dependent checkpoint activation at low nuclei to cytoplasm ratios

**Fig 4:**
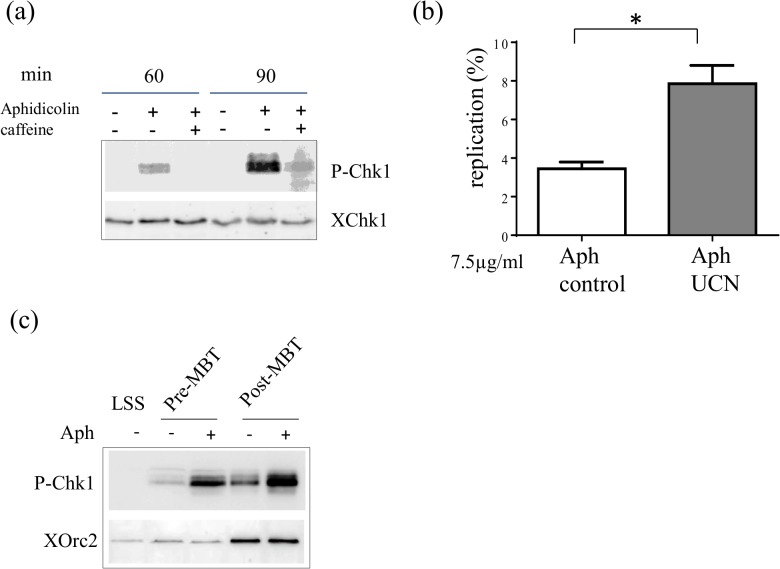
Checkpoint activation upon low nuclei to cytoplasm ratios in the presence of aphidicolin. (a) Sperm nuclei (100 nuclei/μl) were added to egg extracts in the presence of aphidicolin, nuclear extracts were prepared and subjected to gel electrophoresis and western blot analysis using an anti- P-Chk1 and XChk1 antibody, (b) For combing experiments sperm nuclei (100 nuclei/μl) were added to egg extracts in the presence of aphidicolin (7.5 μg/ml), biotin-dUTP and in the presence or absence of UCN-01 (1μM) for 90 min, DNA was isolated and combed, mean replication extent of two independent experiments with SEM (t-test, P = 0.049), (c) *Xenopus* embryos were incubated in aphidicolin (100 μM) for 30 min prior to harvest at stage 8 (pre-MBT) and stage 9 (post-MBT), nuclei extracts were prepared, subjected to gel electrophoresis and western blot analysis using a P-Chk1 antibody and XORC as loading control, LSS (low speed supernatant, extract),* significantly different (P < 0.05).

In *Xenopus* embryos, the DNA content per cell increases rapidly in the absence of transcription during the first 12 cell divisions until the mid-blastula transition (MBT). Chk1 only becomes essential after 12 cell cycles, and is transiently phosphorylated at this stage [22]. We tested whether the replication checkpoint is activated at low nuclei concentration in the *in vitro* system that mimics pre-MBT embryos. Nuclei were incubated at 100 nuclei/μl instead of 2000 nuclei/μl in egg extracts, in the absence or presence of aphidicolin. Proteins of isolated nuclei were analyzed using western blotting. The low nuclei concentration corresponded to 32 cell embryos, about 5 cell cycles after fertilization. We detected strong Chk1 phosphorylation in the presence of aphidicolin, but no signal in its absence (Fig 4A). DNA combing experiments were compared in the presence or absence of Chk1 activity in the presence of aphidicolin. The mean extent of DNA replication (Fig 4B) and the mean fork density (data not shown) in two independent experiments increased in the absence of Chk1 activity. This result shows that the replication checkpoint is activated at low nuclei to cytoplasm (N/C) ratios *in vitro*. We then tested whether Chk1 is phosphorylated in aphidicolin-treated embryos before the MBT. *In vitro* fertilization was performed, and embryos were incubated for 45 min with aphidicolin (100 μM) before nuclear isolation at stage 8 (5 h post fertilization, pre-MBT) or at stage 9 (7 h p.f, post-MBT). Western blot analysis of isolated nuclei rather than whole embryos showed that Chk1 was phosphorylated after replication stress before and after MBT (Fig 4C). We conclude that at low N/C ratios, Chk1 phosphorylation can be detected *in vitro* and *in vivo*, suggesting that Chk1 controls origin activation upon replication stress under these conditions *in vivo*.

### Chk1 inhibition increases fork density during unchallenged S phase

**Fig 5:**
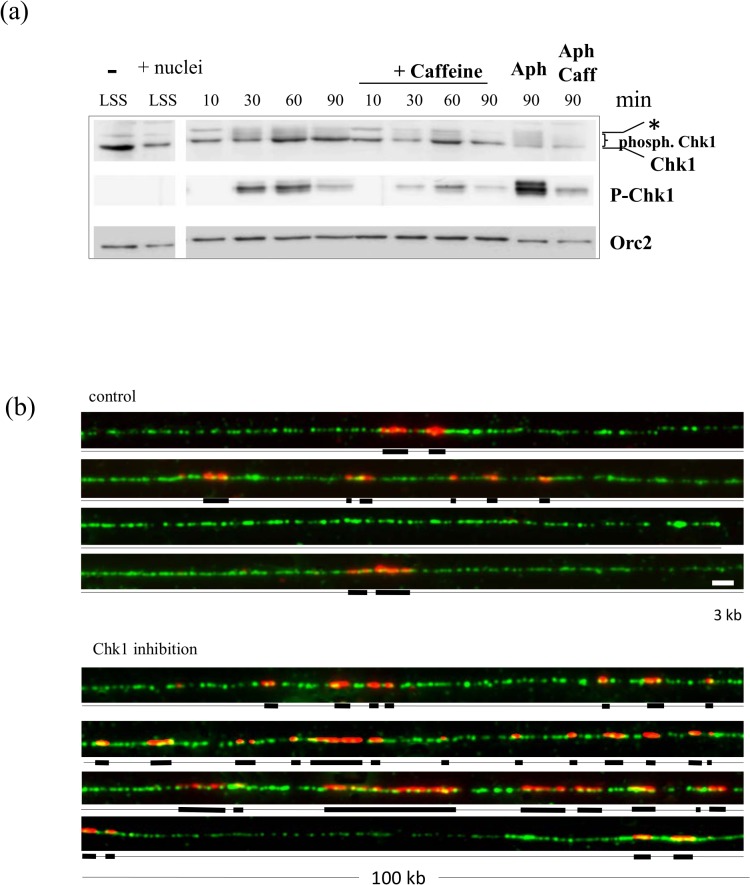
Chk1 activation during unchallenged S phase. (a) Sperm nuclei were added to egg extract for indicated times in the presence or absence of aphidicolin and caffeine, isolated nuclei were subjected to gel electrophoresis and western blot analysis using antibodies against XChk1, anti P-Chk1, XORC2, LSS, low speed supernatant, * marks a non-specific band, (b) Sperm nuclei were added to egg extracts in the presence of Biotin-dUTP for indicated times in the presence or absence of UCN-01 (1μM), Representative combed DNA fibers from early S phase (40 min), in the absence (above) or presence (below) of UCN-01 (merge: green, whole DNA label; red, biotin labeled replication eyes), scale bar 3 kb.

**Fig 6:**
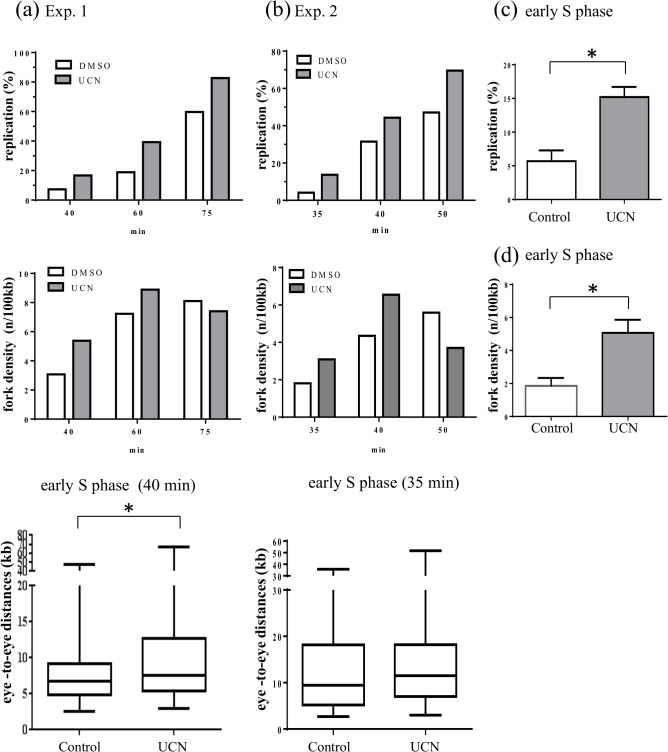
Inhibition of Chk1 induces the increase of fork density but not the decrease of eye-to-eye distances. (a) first independent DNA combing experiment: top: replication extent, middle: fork density (number of forks/100kb), bottom: box-plot of eye-to-eye distances (kb), (b) second independent experiment: top replication extent, middle: fork density (numbers of forks/100kb), bottom: box-blot of eye-to-eye distances, (c) mean replication extent with SEM of four independent experiments from early S phase (t-test, P = 0.0017), (d) mean fork density with SEM of four independent experiments from early S phase (t-test, P = 0.013), * indicates significant difference (P<0.05).

After having observed that only a few induced stalled forks were needed to activate the DNA replication checkpoint, we tested whether Chk1 can regulate origin activation in the absence of external stress, during an unchallenged S phase. In contrast to studies in asynchronous mammalian cells, we use the synchronous *Xenopus in vitro* system that allows us to distinguish temporally distinct events during early, mid and late S phase without synchronization procedures that interfere with checkpoint activation. Sperm nuclei were incubated in egg extracts in the absence of aphidicolin and reactions were stopped at different times for western blot analysis. Chk1 phosphorylation was observed after 30 min, at the onset of replication, and was not observed in controls (extract with or without nuclei incubated on ice for 5 min) (Fig 5A). Chk1 phosphorylation increased in the presence of aphidicolin and was sensitive to the ATM/ATR inhibitor caffeine, as expected. Chk1 phosphorylation during unchallenged S phase has been shown in other studies, although under different experimental conditions [21,45]. Phosphorylated Chk1 was present mainly in nuclear and much less in chromatin bound fractions (S2 Fig), indicating that Chk1 is released from chromatin upon phosphorylation, consistent with results in human cells [46]. In order to analyze origin activation we performed two independent DNA combing experiments using two different egg extracts. Sperm nuclei were incubated in egg extracts in the presence of biotin-dUTP with or without 1 μM UCN-01. The reaction was stopped in early middle or late S phase and DNA was isolated, combed and labeled (Fig 5B). In Fig 6 we show the results of the DNA combing analysis of both experiments separately (a, b) because the replication in experiment 1 was slightly slower than in experiment 2 due to the use of another egg extract. Therefore time points are not identical and not all results cannot be combined and compared directly, especially at later time points. For both experiments replication was accelerated at all time points during S phase in the absence of Chk1 function (Fig 6A, b, top panels). Fork density analysis (Fig 6A and 6B, middle) showed that it strongly increases in early S, less in middle S, and slightly decreased in late S phase in the UCN treated samples. This latter decrease is probably due to more merged eye lengths in the UCN treated sample since we observed an increase in mean eye length (data not shown). Next, we analyzed eye-to-eye distances which we expected to be smaller because fork densities were higher in the presence of UCN. The analysis was performed at the earliest time point in order to avoid replication eye mergers. The comparison of eye-to-eye distance distributions between control and UCN show that either median distances were slightly bigger for experiment 1 at 40 min upon UCN treatment (Fig 6A, bottom, Mann-Whitney test, P = 0.0418) or not significantly different at 35 min (P = 0.398) for experiment 2 (Fig 6B, bottom). Slightly larger eye-to-eye distances in exp.1 could result from more eye mergers due to a small increase in initiations inside clusters after UCN treatment despite an early S phase time point. We combined replication extent and fork density data for early S phase from four independent experiments and found a significant increase of 2.8 and 2.7, respectively (Fig 6C and 6D) after treatment with UCN-01. We conclude that only few additional origins are activated inside already activated clusters but new origins are mainly activated in later clusters upon Chk1 inhibition. These results are therefore in agreement with our aphidicolin data and show that in the absence of external stress, Chk1 also regulates origin activity mainly outside activated replication clusters during S phase. We conclude that after Chk1 inhibition, more origins are activated especially in the beginning of S phase.

**Fig 7:**
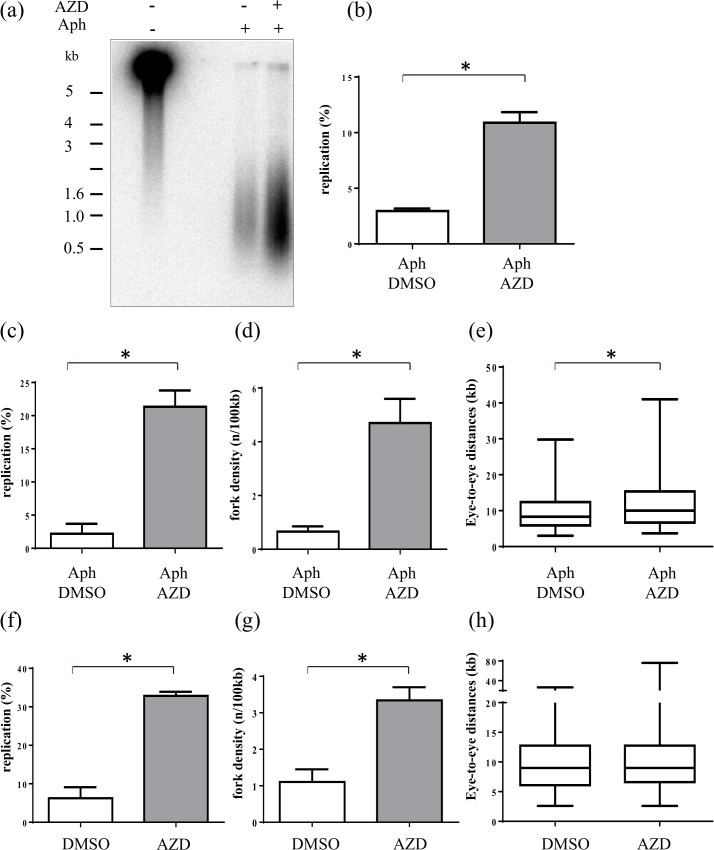
Inhibition of Chk1 activity by AZD-7762 increases DNA synthesis and fork density in the presence and absence of aphidicolin. (a) Sperm nuclei were added to egg extracts in the presence of [α-^32^P]-dATP with or without 0.5 μM AZD-7762 and aphidicolin (7.5 μg/ml) and nascent DNA strands synthesized after 90 min were analyzed by alkaline gel electrophoresis, (b) Quantification of (a) and another independent experiment, mean replication with SEM (t-test, P = 0.013), (c) sperm nuclei were added to egg extracts in the presence of biotin-dUTP, aphidicolin with or without AZD-7762 for 105 min and DNA combing analysis was performed, mean replication extent with SEM of two independent experiments (t-test, P = 0.021), (d) fork density (t-test, P = 0.048), (e) eye-to-eye distances (Mann-Whitney, P = 0.045), (f) sperm nuclei were added to egg extracts in the presence of biotin-dUTP, with or without AZD-7762 and DNA combing analysis was performed, mean replication extent with SEM of two independent experiments at early S phase (t-test, P = 0.013), (g) fork density (t-test, P = 0.046), (h) eye-to-eye distances (Mann-Whitney, P = 0.434), * significantly different (P < 0.05).

In order to confirm the effect of UCN-01, we used a second, more recent Chk1 inhibitor, AZD-7762 [47] in experiments both in the presence and absence of aphidicolin. In the presence of aphidicolin we found in four independent experiments, two nascent strand analysis and two DNA combing experiments, that addition of 0.5μM AZD increased the replication extent in nascent strand (Fig 7A and 7B) and combing analysis (Fig 7C) as observed with UCN-01. This increase was due to a sevenfold higher fork density (Fig 7D) in the presence of AZD. Finally, the distribution of eye-to-eye distances was slightly larger in the presence of AZD in comparison with the control (Fig 7E), but not smaller as expected if origins were activated inside already activated clusters. Furtheron, in the absence of aphidicolin, we found in two independent DNA combing experiments a fivefold increase of replication (Fig 7F) early in S phase which was again due to an increase of fork density (Fig 7G). Distributions of eye-to-eye distances were unchanged as observed after UCN inhibition (Fig 7H). Time course experiments by alkaline DNA gel electrophoresis (S3 Fig) showed that replication extent was still higher at mid and late S phase upon AZD addition. We conclude that Chk1 inhibition by AZD-7762, very similar to UCN-01, results in the activation of replication origins outside but not inside active replication clusters.

### Chk1 overexpression inhibits late replication cluster activation

**Fig 8:**
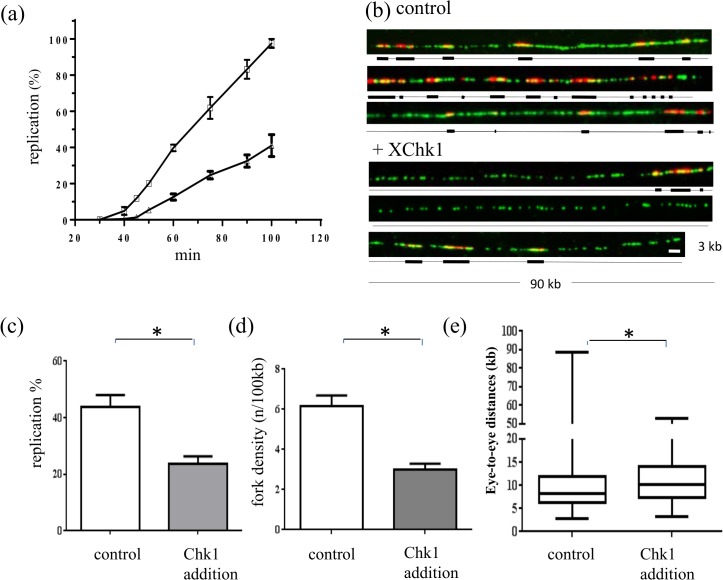
Chk1 overexpression inhibits DNA replication by inhibiting replication cluster activation. (a) Sperm nuclei were added to frozen egg extracts in the presence of [α-^32^P]-dATP for indicated times in the absence (squares) or presence of 120 nM (triangles) recombinant XChk1, DNA was isolated, separated by agarose gel electrophoresis and quantified, mean with SEM of two independent experiments (t-tests: P values <0.05). For combing experiments sperm nuclei were added to egg extracts in the presence of Biotin-dUTP for 45 min in the presence or absence of recombinant XChk1 (120 nM), (b) Representative combed DNA fibers, in the absence (above) or presence (below) of supplemented XChk1 (merge: green, whole DNA label; red, biotin labeled replication eyes), (c) Mean replication extent of two independent experiments with SEM (t-test: P = 0.029), 45 min, (d) Fork density (number of forks/100kb) mean with SEM (t-test: P = 0.037), (e) Box-plot of eye-to-eye distances, 35 min (Mann Whitney, P-value = 0.0002) (kb), Scale bar 3 kb, *significantly different (P < 0.05).

Kumagai *et al*. reported that Chk1 is present in replication competent *Xenopus* egg extracts at a relatively low concentration of 40 nM (2 ng/μl) and that Chk1 overexpression delays mitotic entry. This observation suggested that XChk1 concentration could also be already optimal for DNA replication in the *Xenopus in vitro* system and that overexpression of Chk1 would actually inhibit DNA replication in the absence of external stress. In order to test this hypothesis we produced active recombinant XChk1 (S4 Fig, S5 Fig and S6 Fig), added 120 nM of XChk1 to frozen egg extracts and replicate sperm nuclei in the presence of [α-^32^P]-dATP. The reactions were stopped at indicated time points and DNA was purified. Quantification of DNA synthesis after DNA gel electrophoresis showed a decrease of DNA replication when XChk1 was overexpressed (Fig 8A, S7 Fig). No difference in the timely entry into S phase was detected upon Chk1 overexpression (data not shown). In order to find out how Chk1 addition inhibits DNA replication we performed DNA combing experiments. Sperm nuclei were incubated for 45 min in egg extract the presence of biotin-dUTP and in the absence or presence of 120 nM recombinant XChk1 (Fig 8B). Consistent with the quantification by gel electrophoresis, DNA combing analysis showed that XChk1 addition decreased the percentage of DNA replication in two independent experiments (Fig 8B and 8C) in mid S phase. Next, we analyzed the fork density and found that XChk1 addition decreased more than twofold the number of active forks (Fig 8D). We compared the local eye-to-eye distances in the absence and presence of XChk1 and found a small significant increase upon Chk1 overexpression in one experiment (Fig 8E, median = 8.3 kb control versus 10.1 kb Chk1 addition, Mann Whitney, P-value = 0.0002), but not for a second experiment (S8 Fig) in early S phase. No significant decrease in the median eye length was detected (data not shown) which shows that Chk1 addition did not inhibit elongation. We conclude that Chk1 overexpression in *Xenopus* inhibits DNA replication mainly by inhibition of not activated replication clusters and to a much lesser extent of single origins in already activated clusters.

**Fig 9:**
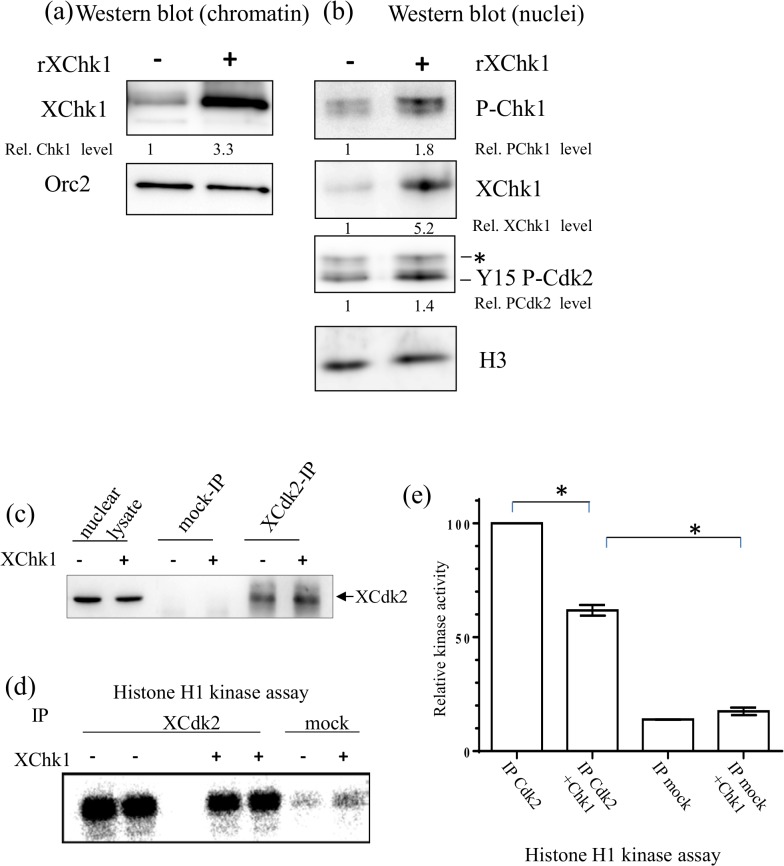
Chk1 overexpression decreases Cdk2 activity. (a) Isolated chromatin and (b) sperm nuclei from reactions in Fig 8B were subjected to gel electrophoresis and western blot analysis using indicated antibodies. Relative protein levels normalized to the loading control Orc2 or histone H3 are indicated below. (c) Western blot for Cdk2 of nuclear lysate (input), mock and Cdk2 IP, (d) Histone H1 kinase assay of cdk2 or mock immunoprecipitates (IP) of nuclear lysates in the absence or presence of recombinant Chk1, duplicates for Cdk2 IPs (e) Quantification of relative histone H1 kinase activity in (d) and two other independent experiments, means of three independent experiments with SEM (t-test: P values < 0.005), * significant difference (P < 0.05).

Next, we wanted to test by which mechanism XChk1 overexpression inhibits replication initiation. Western analysis of chromatin isolated during S phase from replication reactions in the absence and presence of recombinant XChk1 shows that addition of recombinant XChk1 resulted in a threefold increase of chromatin bound XChk1 (Fig 9A). Absolute quantification of chromatin-bound XChk1 using recombinant XChk1 gave an estimate of 700 fg per nucleus before Chk1 addition (data not shown) which corresponds to one Chk1 molecule per replication fork if origins are spaced in average 10kb as reported [33]. Further on, we could detect both an increase of phosphorylated Chk1 and the inhibitory P-Y15-Cdk2 protein levels in nuclei upon Chk1 overexpression (Fig 9B). In order to confirm that Cdk2 activity is actually decreased we performed XCdk2 immuno-precipitations of nuclear extracts prepared from replication reactions in the absence and presence of recombinant XChk1 and tested kinase activity in Cdk2 or mock immunoprecipitations (IP) (Fig 9C) in H1 histone kinase assays (Fig 9D and 9E). The experiment was repeated twice. We found that upon Chk1 addition mean kinase activity of Cdk2-IPs decreased to 61% of the control in a specific manner. We conclude that overexpression of Chk1 in *Xenopus* egg extracts leads to replication cluster inhibition by inhibiting Cdk2 kinase activity.

### Numerical simulation of checkpoint action on origin activation

**Fig 10:**
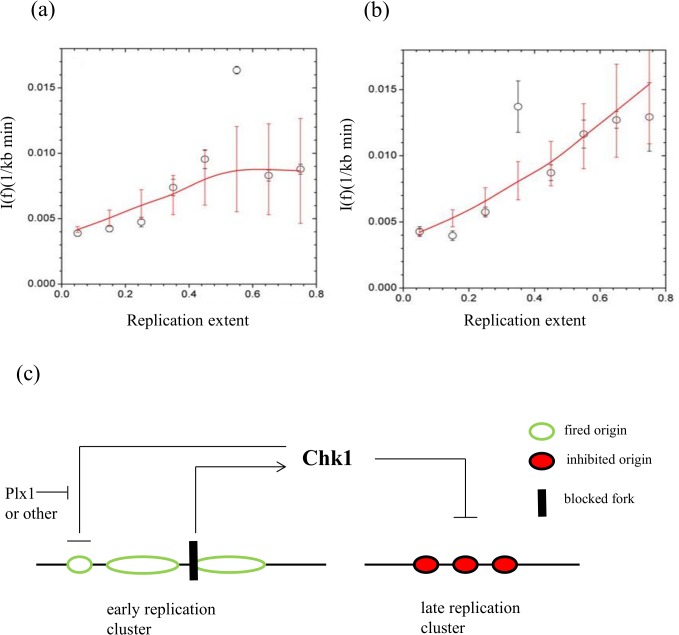
Numerical simulation of initiation frequencies including Chk1 action. Initiation frequency (I*(f)*) increases during early S phase to a higher value after Chk1 inhibition. Experimental (circles) and theoretical, fitted (line) *I(f)* values. (a) in presence of Chk1 activity, (b) in absence of Chk1 activity, see main text for more details, (c) Model of spatio-temporal regulation of origin activation in *Xenopus*, see discussion for details.

In order to better understand the role of Chk1 in the replication program we conducted numerical simulations to recapitulate origin activation including checkpoint pathways, and fitted the results to the experimental DNA combing data. Small replication eyes (1 to 3 kb) from DNA combing data obtained in the presence and absence of Chk1 activity during an unchallenged S phase were used (Figs 5 and 6A), and initiation frequency (*I(f)*) (see Materials and Methods and as described previously [35]) was plotted as a function of replication extent per fiber (Fig 10A, circles). *I(f)* first increased until a plateau was reached after about 45% of replication extent. This plateau was abolished (or maybe reached later) when the checkpoint function was compromised (Fig 10B), as expected from the higher fork density observed in the absence of Chk1 activity. In order to propose a model of replication control that could faithfully reproduce the behavior of *I(f)* in the presence or absence of UCN-01, we built our simulations on our previous model of DNA replication [35] (see Materials and Methods). First, we considered that an encounter between a limiting replication factor and a replication origin will trigger firing with a probability *P*. Second, we added the checkpoint action of Chk1, which inhibits potential replication origins with a temporal probability rate *k*_*Chk1*_. We ran a series of simulations using only these two control variables, but were unable to extract an *I(f)* profile similar to the one extracted from the experimental data. It was previously shown that *Xenopus* Polo-like kinase 1 (Plx1) suppresses the inhibitory action of Chk1 in the presence of aphidicolin [48]. The authors proposed a model in which Plx1 inhibits Chk1 action on replication origins in the neighbourhood of a stalled replication fork. Therefore, we introduced a third, local pathway that blocks the inhibitory action of Chk1 protein over the potential replication origins and assumed that this pathway is also active during an unchallenged S phase. We considered that if a Chk1-inhibited potential replication origin is at a distance *d* of a replication fork, it would have a probability *k*_*polo*_ of recovering its ability to fire. Using this third variable, we found a better match between the *I(f)* extracted from the numerical simulation and the experimental data. We obtained the best fit of *I(f)* with experimental data in the absence of UCN-01 for a probability of inhibition of Chk1 *k*_*Chk1*_ = 0.99 (P < 10^−4^, χ^2^ = 1.03) (Fig 10A, plotted line). This high probability of origin inhibition by Chk1 probably illustrates that regulating the initiation rate by the fork density during a normal, unchallenged S phase is essential. Note that this is also consistent with the observed quantity of Chk1 recruitment onto chromatin (one Chk1 molecule/fork, see above). In the presence of UCN-01, however, we obtained the best fit of *I(f)* with experimental data for a probability of inhibition of Chk1 *k*_*Chk1*_ = 0.3 (Fig 10B, plotted line). This observation suggests that UCN-01 does not completely inhibit Chk1. The initiation rate increases, but is limited by the overall initiation probability and the partial loss of the correlation between fork density and initiation rate. Using combing data from a second independent experiment we obtained very similar results (data not shown). We conclude that to fit our experimental DNA combing data with numerical simulations, we need a combination of two independent means of controlling origin activation: a limiting replication factor and a global checkpoint response but with local checkpoint regulation. These two controls can explain the observed initiation frequencies during S phase in *Xenopus*.

## Discussion

We investigated the role of the checkpoint kinase Chk1 in the replication checkpoint and the spatio-temporal regulation of S phase in the *Xenopus in vitro* system. First, we report that when replication stress is induced by aphidicolin, Chk1 controls chromosomal origin firing in *Xenopus*, consistent with studies in mammalian cells. Second, our experiments demonstrated that during normal, unchallenged S phase and challenged S phase, Chk1 inhibits origin firing at the level of replication clusters, but not within active clusters. Third, we provide the first evidence that modest Chk1 overexpression inhibits DNA replication by inhibiting origin firing in the absence of external replication stress in higher eukaryotes illustrating that Chk1 levels are tightly regulated during normal, unchallenged S phase in higher eukaryotes. Finally, based on fitted mathematical simulations we propose a refined model for spatio-temporal replication program in the *Xenopus* model system showing how Chk1 inhibits late clusters whereas origin firing in early clusters is prohibited by Chk1 inhibition close to activated forks.

### Regulation of replication origin and cluster activation by Chk1 in *Xenopus*

Rad53 inactivation leads to the firing of late replication origins in *S*. *cerevisiae* [11], and Chk1 inhibition by UCN-01 in mammalian cells to the firing of additional origins [49] in the presence of DNA damage or replication stress. Consistent with these results, we found that more replication origins fire in *Xenopus* egg extracts that are replicating nuclei treated with aphidicolin in the absence of Chk1 activity, by inhibiting with two different Chk1 inhibitors or Chk1 depletion. In the absence of induced stress our DNA combing analysis showed that, in the absence of Chk1 activity (Chk1 inhibition by UCN-01 and AZD-7762), 2–3 times more origins fire early in S phase. In an earlier study we also observed an increase of global fork density following ATR inhibition and that *Xenopus* replication origins are organized in clusters that fire at different times during S phase [20]. Combing experiments demonstrated that Chk1 inhibits origins mainly in non-activated replication clusters, but not in already active replication clusters. This differential regulation by the replication checkpoint efficiently inhibits S phase progression, but allows replication of a region with a stalled fork from neighboring origins within an already activated replication cluster.

Several replication clusters are probably present in each cytologically visible replication focus [2]. We previously showed that replication foci number increases early in S phase and decreases late in S phase in *Xenopus* [34]. We tried to investigate foci number in control and UCN-01 treated samples, but single replication foci could not be resolved under these experimental conditions. Upon Chk1 inhibition by UCN-01 or Chk1 depletion, changes in foci patterns or number were detected in chicken DT40 cells during a normal, unchallenged S phase [25] and upon replication stress in human cells [49,50], which illustrates that Chk1 also regulates replication at the level of large chromatin domains. Replication cluster activation has not been addressed in these studies and its organisation is clearly different. Further on, foci activation was studied in the presence of replication inhibitors only. How replication clusters and the larger domains are established and maintained during the cell cycle is still not clear. In *Xenopus*, it probably involves tethering replicons together with different factors such as topoisomerase II [40,51], which might restrict the access of rate limiting initiation factors to later replicating replication clusters. In yeast, forkhead transcription factors Fox2/3 might be needed to tether early origins together [52].

All S phase checkpoint pathways are functional in the *Xenopus in vitro* system, which mimics early developmental stages. However, pre-MBT *Xenopus* embryos exposed to high and prolonged concentrations of aphidicolin continued to divide despite incomplete replication [53], which illustrates the absence of the ATR/Chk1 dependent S-M checkpoint *in vivo*. Therefore it has been proposed that checkpoint activation occurs at the MBT when a critical signal threshold is reached [54]. However, the replication checkpoint is active in the *in vitro* system at a concentration of 1000 nuclei/μl, corresponding to nuclei to cytosolic ratio (N/C ratio) just before the MBT. To confirm that replication can also be activated at low N/C ratios, we reduced the nuclear concentration in the *in vitro* system 10-fold. Even at these very low N/C ratios, the replication checkpoint is activated, as we observed both Chk1 phosphorylation and an increase in fork density, though the checkpoint seems less active at low N/C ratios than at high N/C ratios. In addition, we also detected Chk1 phosphorylation in nuclei from pre-MBT embryos treated with aphidicolin for one cell cycle. These results clearly show that the replication checkpoint can be activated by low N/C ratios *in vitro* and *in vivo*, which challenges the idea that a critical concentration of stalled forks at the MBT is needed to activate ATR and Chk1. Rather than a threshold, we propose that the replication checkpoint shows a gradual response to stalled forks, which is also consistent with its activation during normal, unchallenged S phase [20,21] (our results in this study). These stalled or slowed down forks during unchallenged S phase could arise due to spontaneous DNA damage, a decrease in the optimal concentration of some replication factors or in regions which are difficult to replicate.

A former study did not detect an effect of Chk1 depletion on chromosomal DNA replication in the presence of aphidicolin [23] using an anti-human Chk1 antibody. We speculate that our use of an anti-*Xenopus* antibody or the fact that we used a higher aphidicolin concentration which, as we show, increased the effect of Chk1 inhibition could explain the discrepancy between the studies. While our study was under submission a very recent study showed that inhibition or depletion of Chk1 increases the replication extent of DNA replication during normal S phase in *Xenopus* egg extracts, which is in agreement with our results [55]. However, no combing experiments were performed to show origin and cluster activation upon Chk1 inhibition or depletion.

### Tight Chk1 levels regulate origin activation during normal S phase

In this study we provide the first evidence that modest Chk1 overexpression inhibits DNA replication by inhibiting origin firing in the absence of external replication stress in higher eukaryotes. Our experimental observations are further confirmed by our numerical model which shows that during normal S phase the probability of origin inhibition by Chk1 needs to be already high, in order to fit our experimental combing data. Therefore our results show that the Chk1 activity is negatively rate limiting for DNA replication in the *Xenopus in vitro* system because additional Chk1 inhibits DNA replication. Together with the depletion experiments our study therefore demonstrates that nuclear Chk1 activity needs to be tightly regulated by the cell for proper S phase progression. Loss of one copy of CHK1 causes spontaneous cell death even in the absence of external stress in mammalian cells which the authors interpreted as limiting endogenous Chk1 levels [28]. A recent study reported that expression of one extra-allele of Chk1 in transgenic mice protects against replication stress [56]. The viability of these cells was increased and was associated with a decrease of double strand breaks when transgenic cells were treated with hydroxyurea and aphidicolin. No effect of Chk1 overexpression on BrdU incorporation analyzed by FACS was detected. In *S*. *cerevisiae*, overexpression of a hyperactive allele of the RAD53, the functional CHK1 homologue, is lethal [57]. Our DNA combing experiments show that even in the absence of replication stress three-fold overexpression of Chk1 changes the spatio-temporal program by inhibiting late firing replication clusters mainly. These different effects of Chk1 overexpression could be due to differences in the experimental systems, different levels of overexpression and our more sensitive methods to quantify DNA replication. In mammalian culture cells 20–50% of cellular Chk1 is bound to chromatin [46,58]. In our system, absolute quantification of chromatin bound XChk1 during replication gave an estimate of one chromatin bound Chk1 molecule per active replication fork if one considers that origins are spaced in average 10 kb apart. Upon XChk1 overexpression the nuclear and chromatin bound fraction of Chk1 is increased which leads to the inhibition of DNA replication by downregulation of Cdk2 kinase activity probably via Cdc25A. How exactly Chk1 is recruited to chromatin and the replication fork is not well understood but Chk1 is associated with DNA polymerase alpha [14,59] and the GINS-MCM-Cdc45 helicase [60]. One interesting question is how Chk1 overexpression affects DNA replication if one considers that the amount of Chk1 found on chromatin during normal S phase matches in theory with the presence of one Chk1 molecule per replication fork. A simple explanation would be that supplying additional Chk1 increases the probability of chromatin binding of Chk1 and thereby increasing Chk1 activity on chromatin. Another explanation could be linked to the fact that there are around 20 fold more MCM complexes than ORC complexes bound to the DNA, also known as the MCM paradox [61] which has been shown to be important in dormant origin firing [62]. It is tempting to speculate that the additional Chk1 is recruited to chromatin via excess MCM complexes which then would affect the replication program.

### A model for checkpoint dependent control of DNA replication

Our finding that Chk1 mainly regulates origins distal to already activated replication clusters suggests that mechanisms exist that prevent Chk1 from inhibiting potential origins in clusters that already contain activated origins. In *Xenopus*, depletion of Polo-like kinase 1 leads to an increase in Chk1 phosphorylation and a decrease in both Cdk2 activity and Cdc45 loading in response to replication stress [48]. Costanzo and co-workers proposed a model suggesting that Plx1 plays a role in checkpoint adaptation by inhibiting checkpoint action close to stalled forks but they did not analyze replication origin and cluster activation in detail by DNA combing. Different scenarios for local/global regulation of origin firing have been suggested for cells under replication stress or DNA damage [63], but that could also apply to a normal S phase.

In a previous study, based on DNA combing experiments with intact checkpoint pathways, we proposed a model for the control of DNA replication in *Xenopus* [35]. This model combines time-dependent changes in the availability of a replication factor and a fork-density dependent affinity of this factor for potential origins to explain the observed increase in the initiation rate and fork density in our system. But the inhibitory regulation by the replication checkpoint had not been included. Here, we refine this model by adding a Chk1-dependent inhibition of origins combined with local repression of this inhibition in the proximity of previously fired origins (Fig 10C). This local repression could be mediated by Plx1 and/or by unknown factors. The Chk1-dependent control together with a control implying a limiting replication factor best matches the experimental initiation frequencies (*I(f)*). This differential regulation of the checkpoint pathway would allow the cell to replicate a replication cluster with a stalled fork, which might not be able to resume from neighbor origins. But globally replication is slowed down until the replication stress disappears. It would be interesting to test whether these pathways could also explain initiation rates in mammalian systems.

In conclusion, our study demonstrates that both a highly active Chk1-dependent replication checkpoint and rate limiting initiation factors are needed for the sequential activation of replication clusters in *Xenopus* egg extracts, which explains the essential role of Chk1 in regulating origin firing and genome stability during S phase. Thus, this basal replication checkpoint activity is an efficient way for cells to adapt the optimal replication fork density to the concentration of replication factors during S phase.

## Supporting Information

S1 Fig Eye-to-eye distance distribution does not significantly change upon Chk1 inhibition by UCN in the presence of aphidicolin. Box-plot of eye-to-eye distances (ETED), second independent experiment, control DMSO, UCN addition, 90 min Aphidicolin (Mann-Whitney Test, P = 0.3702).
(PDF)

S2 Fig Phospho-Chk1 is not bound to chromatin. Sperm nuclei were added to egg extracts for the indicated times, nuclear extracts or chromatin fractions were subjected to gel electrophoresis and western blot analysis using antibodies against anti P-Chk1, XORC2.
(PDF)

S3 Fig Time course of replication upon AZD addition. Sperm nuclei were added to egg extracts in the presence of [α^32^P]-dATP, replication was stopped at indicated times, purified DNA was subjected to gel alkaline electrophoresis and replication quantified on a phosphorimager with 90 min AZD time point as 100%, mean with SEM of two independent experiments.
(PDF)

S4 Fig Production of recombinant XChk1. Recombinant XChk1 was purified from Baculovirus-infected insect cells His-tagged XChk1 after purification with Nickel-Sepharose loaded on a 10% polyacrylamide gel and Coomassie stained. Lanes: 1. Protein Marker, 2. 10 μl XChk1-6His (0.2mg/ml).
(PDF)

S5 Fig Production of anti-XChk1 antibody. Anti-XChk1 antibody made against full length XChk11 recognizes recombinant XChk1 and endogenous XChk1, Lanes: 1. Recombinant 6His-XChk1, 2. S phase Xenopus egg extract,* marks non-specific band.
(PDF)

S6 Fig Chk1 kinase assay. CHKtide kinase assay, recombinant Chk1 was incubated with or without a specific Chk1 substrate CHKtide in the presence of [γ^32^P]-ATP for 30 min at 30°C, separated on 15% SDS polyacrylamide gel, dried and analyzed on a phosphoimager.
(PDF)

S7 Fig Effect of Chk1 overexpression on DNA replication. Sperm nuclei were replicated in egg extract in the presence ofα^32^P]-dATP, replication was stopped at indicated times, purified DNA was subjected to agarose electrophoresis.
(PDF)

S8 Fig Eye-to-eye distance distribution of second independent DNA combing experiment in absence and presence of recombinant Chk1, 45 min (Mann-Whitney, P = 0.296). (PDF)

S1 File Raw DNA combing data from Figs 3, 4, 6, 7 and 8. (ZIP)
