# Supplementary figures and images for: Tight Chk1 Levels Control Replication Cluster Activation in Xenopus
Source: PLoS One. 2015 Jun 5;10(6):e0129090. doi: 10.1371/journal.pone.0129090 (PMC4457610; doi:10.1371/journal.pone.0129090)

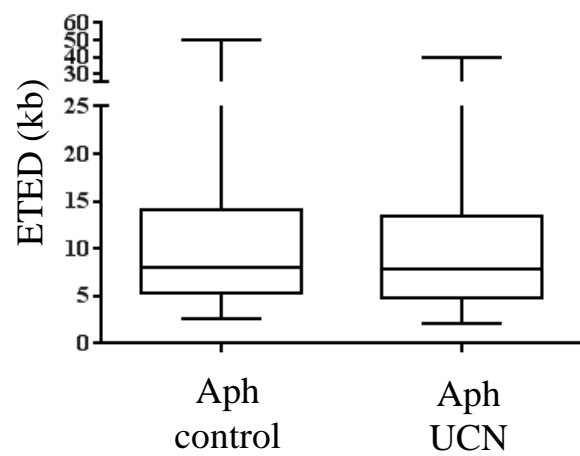

Supplement: S1 Fig — Box-plot of eye-to-eye distances (ETED), second independent experiment, control DMSO, UCN addition, 90 min Aphidicolin (Mann-Whitney Test, P = 0.3702). (PDF) [file pone.0129090.s001.pdf]

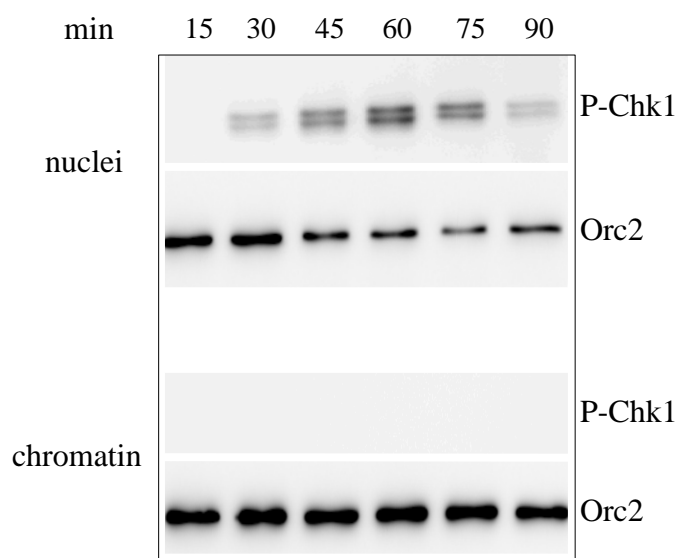

Supplement: S2 Fig — Sperm nuclei were added to egg extracts for the indicated times, nuclear extracts or chromatin fractions were subjected to gel electrophoresis and western blot analysis using antibodies against anti P-Chk1, XORC2. (PDF) [file pone.0129090.s002.pdf]

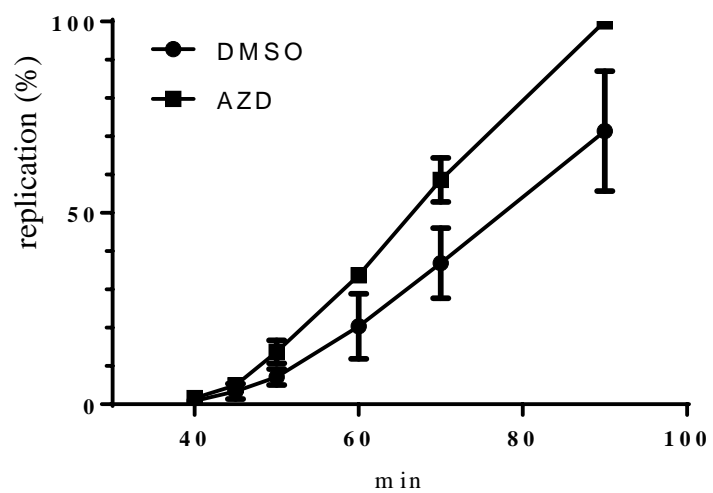

Supplement: S3 Fig — Sperm nuclei were added to egg extracts in the presence of [α32P]-dATP, replication was stopped at indicated times, purified DNA was subjected to gel alkaline electrophoresis and replication quantified on a phosphorimager with 90 min AZD time point as 100%, mean with SEM of two independent experiments. (PDF) [file pone.0129090.s003.pdf]

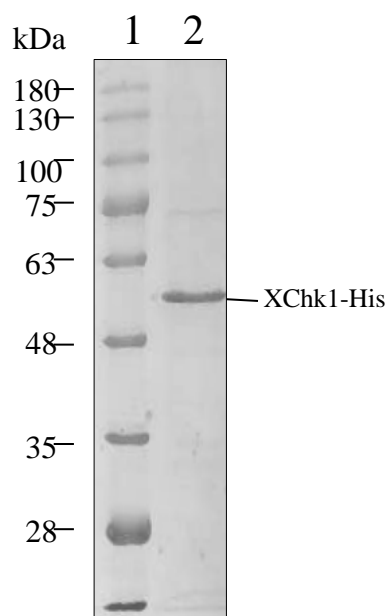

Supplement: S4 Fig — Recombinant XChk1 was purified from Baculovirus-infected insect cells His-tagged XChk1 after purification with Nickel-Sepharose loaded on a 10% polyacrylamide gel and Coomassie stained. Lanes: 1. Protein Marker, 2. 10 μl XChk1-6His (0.2mg/ml). (PDF) [file pone.0129090.s004.pdf]

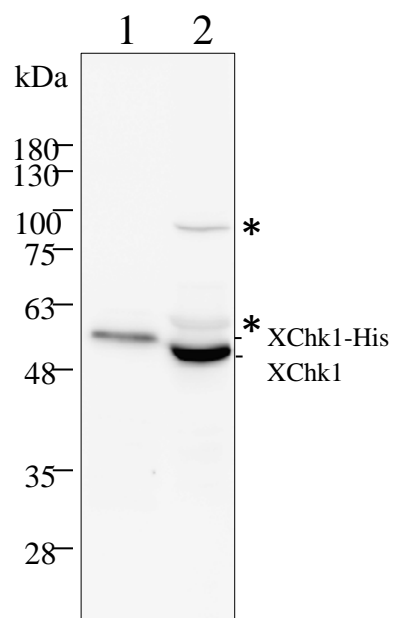

Supplement: S5 Fig — Anti-XChk1 antibody made against full length XChk11 recognizes recombinant XChk1 and endogenous XChk1, Lanes: 1. Recombinant 6His-XChk1, 2. S phase Xenopus egg extract,* marks non-specific band. (PDF) [file pone.0129090.s005.pdf]

|         |   |   |   |    |   |    |
|---------|---|---|---|----|---|----|
| Chk1    | + | - | + | +  | + | +  |
| CHKtide | - | + | + | ++ | + | ++ |
| UCN-01  | - | - | - | -  | + | +  |

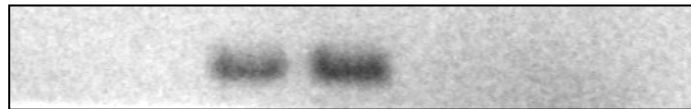

Supplement: S6 Fig — CHKtide kinase assay, recombinant Chk1 was incubated with or without a specific Chk1 substrate CHKtide in the presence of [γ32P]-ATP for 30 min at 30°C, separated on 15% SDS polyacrylamide gel, dried and analyzed on a phosphoimager. (PDF) [file pone.0129090.s006.pdf]

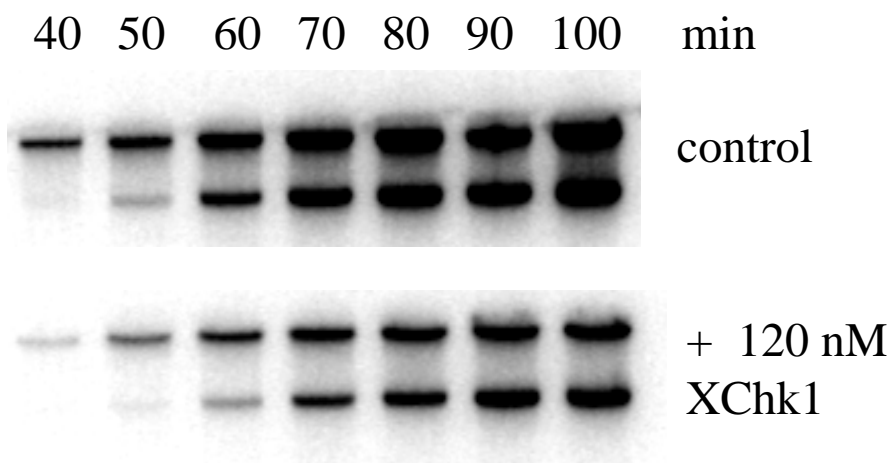

Supplement: S7 Fig — Sperm nuclei were replicated in egg extract in the presence ofα32P]-dATP, replication was stopped at indicated times, purified DNA was subjected to agarose electrophoresis. (PDF) [file pone.0129090.s007.pdf]

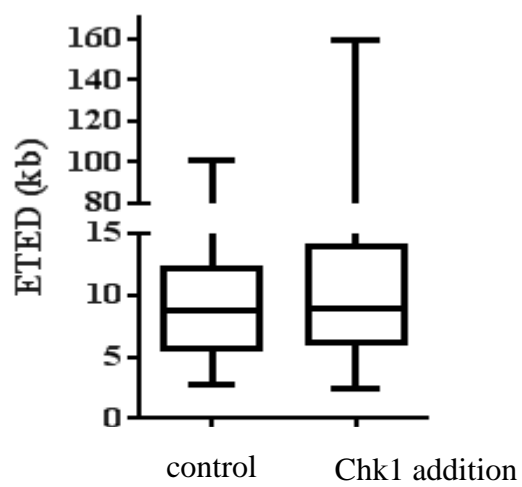

Supplement: S8 Fig — (PDF) [file pone.0129090.s008.pdf]
